# Using anti‐Aβ antibodies to modulate the multi‐pronged human microglia response to Aβ pathology

**DOI:** 10.1002/alz.087213

**Published:** 2025-01-03

**Authors:** Giulia Albertini, Magdalena Zielonka, Marie‐Lynn Cuypers, Veerle van Lieshout, An Snellinx, Emanuela Pasciuto, Leen Wolfs, Katleen Craessaerts, Katrien Horré, Tom Jaspers, Mark Fiers, Maarten Dewilde, Bart De Strooper

**Affiliations:** ^1^ Flanders Institute for Biotechnology (VIB), Leuven Belgium; ^2^ KU Leuven, Leuven Belgium; ^3^ Flanders Institute for Biotechnology (VIB), Antwerp Belgium; ^4^ University of Antwerp, Antwerp Belgium; ^5^ UK Dementia Research Institute, University College London, London United Kingdom

## Abstract

**Background:**

While social and medical debate about the efficacy and safety of anti‐Aβ immunotherapy is ongoing, one thing that emerged is that we have little understanding of the working mechanisms of these antibodies and this lack of knowledge complicates the interpretation of the clinical results. Here, we aimed to establish if microglia are required for the efficacy of Lecanemab, one of the most promising FDA‐approved disease‐modifying therapy for AD (Van Dyck et al. N Engl J Med 2023).

**Method:**

To do so, we crossed App^NL‐G‐F^ mice with Csf1r^ΔFIRE/ΔFIRE^ mice (Rojo et al. Nat Commun 2019) to generate mice that show key features of Aβ pathology but genetically lack mouse microglia. We then assessed the effect of Lecanemab treatment on Aβ load and neuritic dystrophy.

**Result:**

We demonstrate that Lecanemab lacks efficacy in the absence of microglia. On the other hand, when we xenotransplant human microglia into the brain of these mice (as described in Mancuso et al. Nat Neurosci 2019), we show that Lecanemab treatment significantly ameliorates both Aβ load and neuritic dystrophy. Furthermore, by employing scRNAseq on sorted human microglia, we demonstrate that Lecanemab treatment affects the transcriptome of the microglia by inducing a number of genes related phagocytosis, interferon response and immune activation. Functionally, we also established that Lecanemab‐treated human microglia ingest more amyloid‐β *in vivo*.

**Conclusion:**

Overall, we provide the first evidence that microglia are crucial for the efficacy of anti‐Aβ immunotherapy and provide real insight into the working mechanisms of this first disease‐modifying therapy for AD.

